# COVID-19 Spread in Saudi Arabia: Modeling, Simulation and Analysis

**DOI:** 10.3390/ijerph17217744

**Published:** 2020-10-23

**Authors:** Hend Alrasheed, Alhanoof Althnian, Heba Kurdi, Heila Al-Mgren, Sulaiman Alharbi

**Affiliations:** 1 Department of Information Technology, College of Computer and Information Sciences, King Saud University, Riyadh 11451, Saudi Arabia; aalthnian@ksu.edu.sa; 2 Department of Computer Science, College of Computer and Information Sciences, King Saud University, Riyadh 11451, Saudi Arabia; hkurdi@ksu.edu.sa; 3 Department of Mechanical Engineering, School of Engineering, Massachusetts Institute of Technology, Cambridge, MA 02142, USA; 4 Department of Software Engineering, College of Computer and Information Sciences, King Saud University, Riyadh 11451, Saudi Arabia; heila.almogren@gmail.com; 5 Department of Botany and Microbiology, College of Sciences, King Saud University, Riyadh 11451, Saudi Arabia; sharbi@ksu.edu.sa

**Keywords:** COVID-19, simulation model, network-based epidemic model, contact network, node susceptibility

## Abstract

The novel coronavirus Severe Acute Respiratory Syndrome (SARS)-Coronavirus-2 (CoV-2) has resulted in an ongoing pandemic and has affected over 200 countries around the world. Mathematical epidemic models can be used to predict the course of an epidemic and develop methods for controlling it. As social contact is a key factor in disease spreading, modeling epidemics on contact networks has been increasingly used. In this work, we propose a simulation model for the spread of Coronavirus Disease 2019 (COVID-19) in Saudi Arabia using a network-based epidemic model. We generated a contact network that captures realistic social behaviors and dynamics of individuals in Saudi Arabia. The proposed model was used to evaluate the effectiveness of the control measures employed by the Saudi government, to predict the future dynamics of the disease in Saudi Arabia according to different scenarios, and to investigate multiple vaccination strategies. Our results suggest that Saudi Arabia would have faced a nationwide peak of the outbreak on 21 April 2020 with a total of approximately 26 million infections had it not imposed strict control measures. The results also indicate that social distancing plays a crucial role in determining the future local dynamics of the epidemic. Our results also show that the closure of schools and mosques had the maximum impact on delaying the epidemic peak and slowing down the infection rate. If a vaccine does not become available and no social distancing is practiced from 10 June 2020, our predictions suggest that the epidemic will end in Saudi Arabia at the beginning of November with over 13 million infected individuals, and it may take only 15 days to end the epidemic after 70% of the population receive a vaccine.

## 1. Introduction

Coronavirus, a genus of the Coronaviridae family, are enveloped viruses with a large plus-stranded RNA genome. The genomic RNA is 27–32 kb in size and is capped and polyadenylated. Three serologically distinct groups of coronaviruses have been described, with viruses in each group characterized by their host range and genome sequence. Coronaviruses belong to a large family of viruses known to cause illnesses ranging from the common cold to more severe diseases, such as Middle East Respiratory Syndrome (MERS) and Severe Acute Respiratory Syndrome (SARS). A novel coronavirus, SARS-Coronavirus-2 (SARS-CoV-2) was identified in December 2019 in Wuhan, China, as a coronavirus that had not been previously identified in humans; this novel coronavirus is also known as the Coronavirus Disease 2019 (COVID-19).

Since its identification, SARS-CoV-2 has spread rapidly, affecting over 200 countries and causing the 2019/2020 coronavirus pandemic. It was declared as a Public Health Emergency of International Concern on 30 January 2020 by the World Health Organization (WHO). To date, many countries and regions have implemented lockdown measures and strict social distancing to limit the propagation of the virus. From a strategic and healthcare management perspective, the propagation pattern of the disease and the prediction of its spread over time is of great importance, which can save lives and minimize the social and economic consequences. Epidemiological modeling is a powerful tool that can help understand disease spread, control, and prevention. Different mathematical epidemic models have been used in the literature, including statistical models [1], mathematical models [2,3,4,5,6,7,8,9,10], and network-based models [11,12,13].

Mathematical epidemic models are used to predict the course of an epidemic and develop methods for controlling it by comparing different possible scenarios based on the observed data. One of the widely used models is the Susceptible-Infected-Recovered (SIR) model [14,15], where individuals are assigned into three compartments, i.e., susceptible (S), infected (I), and recovered (R). Each individual belongs to one compartment and changes his/her state over time. An individual can transition from susceptible to infected with a specific infection rate. Each individual can also transition from infected to recovered according to a specific recovery rate. This simple epidemic model works well for a homogeneous population that exhibits similar contact patterns, with contact probabilities between any two individuals considered to be equal. However, recent research has shown that the contact patterns in a real population are heterogeneous [16].

As bidirectional social contacts are key factors in disease spreading, modeling epidemics on contact networks has been increasingly used to understand disease transmission and evaluate the impact of potential disease control [17,18,19]. This is because contact relationships between individuals that allow infection propagation naturally define a network. Hence, understanding the contact network structure can improve the predictions of the infection distribution among individuals and allow the simulation of the full epidemic dynamics. Networks allow the modeling and simulation of disease control measures by manipulating the connections among different individuals.

In this work, we propose a simulation model for the spread of COVID-19 in Saudi Arabia using a network-based SIR epidemic model. We first generated a contact network that captured the realistic social behaviors and dynamics of individuals in the population of Saudi Arabia. We aimed to match the model simulations with empirical data and then used the model to evaluate the effectiveness of the control measures employed by the Saudi government, to predict the future dynamics of the disease in Saudi Arabia according to different scenarios, and to predict the percentage of individuals that must be vaccinated to stop the outbreak (when a vaccine becomes available). Modeling the spread of COVID-19 in Saudi Arabia has been discussed in the literature [20,21,22,23]; however, no studies used a network-based model that captured the social and dynamic properties that are intrinsic to Saudi society. Further, control measures, such as school closures, mosque closures, domestic flight shutdowns, and curfews, were not considered.

The proposed model is used to explain how social measures, such as social distancing and regional lockdowns, influence the model parameters, which, in turn, change the number of infected cases over time. The proposed model considers the dynamic nature of individual contact behaviors and the variations in susceptibility and infectivity between individuals. The main contributions of the work can be summarized as follows:

1. We built a model for contact networks that captures the social properties and dynamics intrinsic to Saudi Arabia’s society. A set of attributes was defined for each node (representing each individual), including age, gender, nationality, and location. This is important as network structure and node attributes are crucial factors in the COVID-19 epidemic spreading process.
2. We built a network simulation model of the spread of COVID-19 in Saudi Arabia using the widely adopted SIR model. Using our network simulations, we analyzed the processes by which COVID-19 spreads.
3. We analyzed the effectiveness of the response of Saudi authorities using our network simulations.
4. We predicted the future dynamics of the disease in Saudi Arabia under different scenarios.
5. We investigated the effectiveness of different vaccination strategies.

In this work, we evaluated the effectiveness of Saudi Arabia’s control measures on the epidemic dynamics. Our results showed that strict local control measures, such as school closures, mosque closures, and flight shutdowns, play an important role in controlling the spread of the disease. In particular, mosque closures have the greatest impact on decreasing the transmission rate of the disease. Our key results are in agreement with previous findings in China [11,24,25] and in the United States [11]. Our model suggests that Saudi Arabia would have faced the peak of the outbreak on 21 April 2020 with a total of about 26 million infections if it had not imposed the control measures. This illustrates the importance of employing strict measures for flattening the epidemic curve of the infection and reducing the size of the epidemic. The strict social measures delay the peak of infection and minimize its period. Altogether, these effects limit the burden on the healthcare system and prevent it from being overwhelmed.

We also predicted the future dynamics of the outbreak in Saudi Arabia for the upcoming six months using multiple scenarios. According to the current data, the proposed model suggested that the peak would be roughly at the beginning of July, reaching a peak of 0.5% of the population if people did not practice strict social distancing. The peak represents the highest number of daily infections.

Using our simulations, we also computed the percentage of people that must be vaccinated to stop the epidemic. Our results suggest that the outbreak can be contained by increasing the percentage of the vaccinated population (but without resorting to mass vaccination of the population). According to our results, the proposed simulation model provides insights that reflect the dynamic behavior of COVID-19 under different scenarios. The results can guide the local healthcare system for making decisions during the critical periods of the epidemic.

The rest of the paper is organized as follows. In Section 2, we discuss related literature works, and, in Section 3, we describe the method, including the contact network generation model, the data, and the network simulation model. In Section 4 and Section 5, we present and discuss the simulation results. Finally, Section 6 concludes the work.

## 2. Literature Review

The epidemic progression of COVID-19 has received increased attention from the research community since its outbreak in late 2019. The importance of understanding the virus transmission dynamics and further predicting the epidemic curve for public policy healthcare control measures has prompted multiple modeling efforts to control the outbreak [26,27]. Existing contributions in the epidemiological modeling of COVID-19 include different types of models, such as statistical models [1], mathematical models [2,3,4,5,6,7,8,9,10], network-based models [11,12,13], and phenomenological models [28]. Due to their conceptual and mathematical simplicity, mathematical models, especially SIR compartmental models, have long been popular in modeling epidemic dynamics [29,30].

An SIR model describes the spread of a disease in a population, where individuals are assigned into three compartments: susceptible (S), infected (I), and recovered (R) [14,15]. However, previous studies [16] reported that compartmental models lack explicit modeling of contact structures among individuals, which play a crucial role in understanding and modeling the dynamics of the spread of directly transmissible diseases. Compartmental models assume homogenous mixing, where all individuals are equally likely to encounter infection, which may not reflect reality [31,32,33]. Manzo [16] argued that a major problem with these kinds of compartmental models is that they can only be used with population-wide interventions because they do not model the topology of realistic social interactions.

For these reasons, network-based models have been considered as an alternative for the epidemiological modeling of directly transmissible diseases [17,18,19]. In such models, an infection may only spread over an arc between two nodes (or individuals) in the network that represents a contact. In the literature, several studies have addressed the deficiencies of previous compartmental models by extending SIR-type models on a generated contact network [19]. For instance, Salathe and Jones [34] adopted this approach to study the effect of community structure on the epidemic dynamics of infectious disease and immunization intervention. Volz [35] modeled SIR dynamics on a static random network, which represents the population structure of susceptible and infected individuals and their contact patterns with an arbitrary degree distribution. The authors extended their work in [36] to cover a dynamic random network because contact patterns are inherently dynamic such that individuals tend to make and break relationships over time.

Miller et al. [30] proposed an edge-based compartmental model, which unlike compartmental models, assumes a heterogeneous contact rate and considers the partnership duration. Read and Keeling [37] investigated how local or global transmission routes in a contact network may affect the evolutionary selection of the transmission rate and infectious period, which determines the transmission dynamics of infectious diseases. Ball et al. [38] proposed a stochastic SIR network epidemic model with preventive dropping, where a susceptible individual can practice social distancing by removing its edge to an infectious individual. Due to the importance of social mixing patterns on modeling epidemic dynamics and evaluating the employed control measures, many research efforts have been made to estimate the patterns in different countries [39,40,41,42].

Despite the success of network-based models, several published studies on COVID-19 modeling, including those supporting policy decision making, have focused on compartmental models [2,3,4,5,6,7,8,9,10,20,21,22,23,43]. Manzo [16] urged researchers to direct their efforts toward network-based SIR models and to start discussing a large-scale collection of empirical network data to foster such models. Ferguson et al. [13] used a network-based model to study the impact of non-pharmaceutical interventions on reducing the spread of COVID-19 to advise policymaking in the UK and other countries. The authors adopted an individual-based simulation model published in [44,45], where spatial details were included, such as the household, school, workplace, and the wider community. The authors used real data to define multiple attributes of the model, including age and household distribution size, average class sizes, staff-student ratios, and workplace size.

Peirlinck et al. [11] evaluated the effectiveness of intervention strategies and predicted the outbreak peak in China and the US. The authors modeled the COVID-19 outbreak dynamics by combining a network model, where the nodes represent states and the edges represent connections between them, and an epidemic susceptible (S), infected (I), exposed (E), and recovered (SEIR) model. In their study, Liu et al. [12] developed a contact network and a model without contact to simulate the unfortunate incident of the COVID-19 outbreak in the Diamond Princess cruise ship in two stages. The first stage was unprotected contact and the second stage was divided into two scenarios: protected contact and airborne spread of the virus. The authors designed a small-world network-based chain-binomial model [46,47] for the unprotected contact stage, a contact network epidemic model for protected contact for the crew stage, and a no-contact susceptible and infected model (NCSI) for the airborne spread for the passenger stage. They used Bayesian inference and Metropolis-Hastings sampling to estimate the model parameters.

Several existing contributions modeled the COVID-19 outbreak in Saudi Arabia using different models [20,21,22,23]. For instance, Alboaneen et al. [20] predicted that Saudi Arabia would have a maximum total cases of 79,000 using logistic growth and SIR models. In [21], Alharbi et al. found that the SIR model provided the best fit to the data compared to the generalized logistic, Richards, and Gompertz models. Their results predicted that the total number of infected cases would reach 359,794 and that the pandemic would end by early September 2020. Aletreby et al. [22] predicted that the pandemic would peak by the end of July 2020. Further, the work in [23] used the SIR model to predict future trends and compare the impact of control measures taken by Saudi Arabia and the United Kingdom on the outcomes of COVID-19 pandemic. Their results indicated that early extreme measures imposed by the Saudi authority played a major role in reducing the spread of the disease, compared to the UK.

Although there are some contributions that discussed COVID-19 in Saudi Arabia [48,49,50,51,52] and others modeled the epidemic dynamics of the COVID-19 outbreak in the country using different models, such as SIR [20,21,23], SEIR [22], logistic growth [20], and generalized logistic, Richards, and Gompertz models [21], none have used a network-based model or considered the social properties and dynamics intrinsic to Saudi Arabia’s society. Control measures, such as school closures, mosque closures, domestic flight shutdowns, and curfews, were not considered. This work seeks to fill that gap by investigating the spread of COVID-19 in Saudi Arabia using a network-based epidemic simulation model.

## 3. Method

The first positive COVID-19 case in Saudi Arabia was confirmed on 2 March 2020 with more cases sporadically appearing in the following few weeks [53,54,55]. According to the Saudi Ministry of Health [56], the vast majority of infected people were home-comers from high-risk regions and their immediate contacts [53,57,58].

The proposed simulation is a stochastic discrete network-based model that explicitly represents individuals and their interactions. First, we created a synthetic contact network that matches the essential structural properties of Saudi Arabia’s society. The synthetic population was constructed to statistically match the population demographics of Saudi Arabia. Secondly, we modeled the spread of COVID-19 in Saudi Arabia using a classic SIR model. Finally, we conducted the contact network generation, simulation, and all analyses using the Python-based Networkx library [59]. The generated network dataset, model parameters, and population demographics data are all available at https://github.com/halrashe/COVID-19_SA_Simulation. 

### 3.1. Contact Network Generation

**Figure A1:**
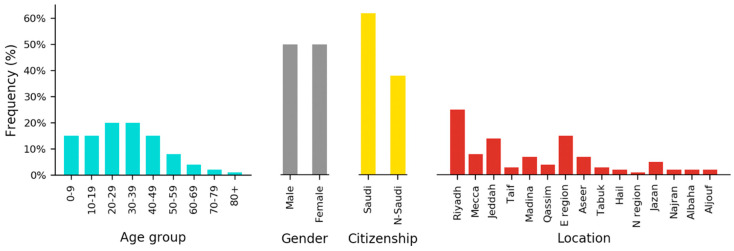
Distribution of nodes by age group, gender, citizenship, and location.

To simulate the spread of COVID-19 in Saudi Arabia, we generated a contact network using the intrinsic properties and dynamics of Saudi Arabia’s Society. We preserved the Saudi-related demographics and social features that are essential for the transmission of infection. Therefore, our network generation model captures key individual and social aspects. First, the network captures the properties of individuals by assigning a set of attributes to each node, including age group, gender, citizenship, and location. Secondly, the network conforms to several observed contact behaviors among individuals such as location and age assortativity [60]. We used data from the Saudi General Authority for Statistics [61] to assign the distribution of individuals for each attribute [62,63] (see Figure A1 and Table A1 in the Appendix).

**Figure 1:**
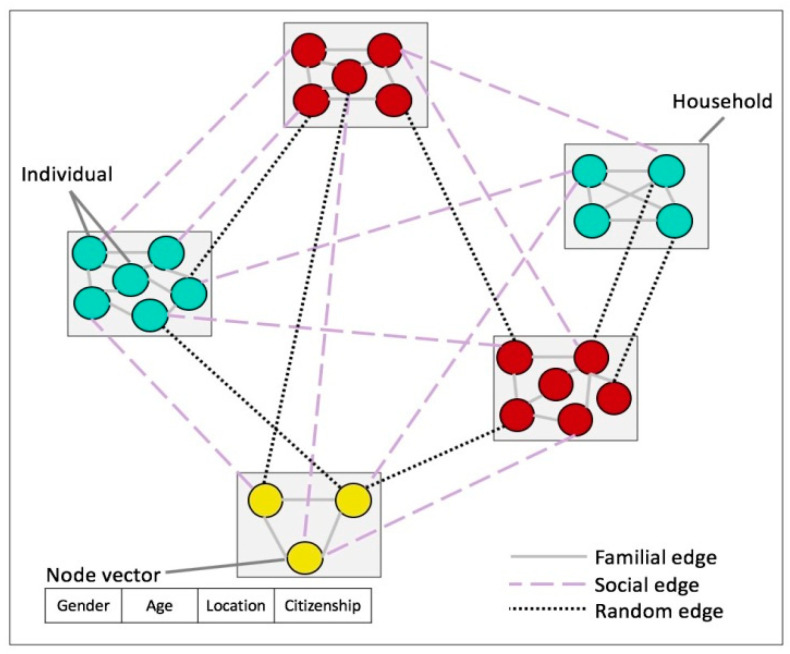
Schematic representation of the three edge types in the contact network.

This is computationally challenging [32,64]; therefore, a contact network with a population of *N* = 10,500 individuals was generated with given age group, gender, citizenship, and location distributions. The geographic locations used to construct the network corresponded to the 15 main administrative regions in Saudi Arabia. Node connections represent contacts that may take place before and during the period of the epidemic. Three connection types between node pairs were used in the network: familial, social, and random. See Figure 1 for a schematic of the network.

We define our undirected and unweighted contact network *G* = (*V,E*), where *V* represents the set of individuals in the population and *E* represents the contact relationships between them. In the contact network *G*, each individual belongs to a household and the household sizes correspond to the values for Saudi Arabia reported in [65]. Each household is represented as a complete graph in which every node is connected to every other node by a familial edge. Nodes from two different households can be linked in two ways, i.e., based on similarity (social edges) or at random (random edges).

Nodes are linked with social edges with a probability proportional to their similarity (i.e., a higher node similarity implies a higher chance of connection in the contact network). Two nodes are considered similar when they exhibit similar attributes. The similarity of two nodes *u* and *v*, denoted as *similarity*(*u,v*), is computed using the scaled Euclidean distance between the two node vectors based on their attributes. Let *U* and *V* be the vectors corresponding to nodes *u* and *v*. We first construct the vectors of the two nodes (the vector length is equal to the number of attributes describing each node, which is 4 in this case). The corresponding elements in both vectors have values of 0 and 1, respectively, if the two nodes have a different value for an attribute. Otherwise, the corresponding elements of the two vectors have a value of 0. Then, the similarity is computed as follows:

(1) $$similarity\left(u,v\right)=\;\sqrt{a{\left(U_{0}-V_{0}\right)}^{2}+a{\left(U_{1}-V_{1}\right)}^{2}+a{\left(U_{2}-V_{2}\right)}^{2}+c{\left(U_{3}-V_{3}\right)}^{2}}$$

where *a* and *c* are constants such that 3*a* + *c* = 1 and *c >> a*. The goal here is to assign the location attribute a larger weight because it plays the most important role in deciding the contact relationship among node pairs. If two nodes are not similar, then they may be connected randomly with a probability of *p*^+^*_loc_* if they both belong to the same location and with a probability of *p*^+^*_random_* if they belong to different locations (*p*^+^*_random_ << p*^+^*_loc_*).

Each edge *e_u,v_* connecting node *u* and *v* has a type attribute describing its formation. Here, we use three edge types. The first one is *familial* when the two nodes *u* and *v* belong to the same household. The second is *social* when *e_u,v_* is formed as a result of the similarity between *u* and *v*. The third one is *random* when *e_u,v_* is formed completely at random. Social edges represent contact relationships as a result of sharing school, work, interests, and neighborhoods. Random edges represent contact relationships that occur as a result of coming into contact with another individual in a public place, a taxi, an airport, etc., or due to social contact that is not based on similarity.

**Figure 2:**
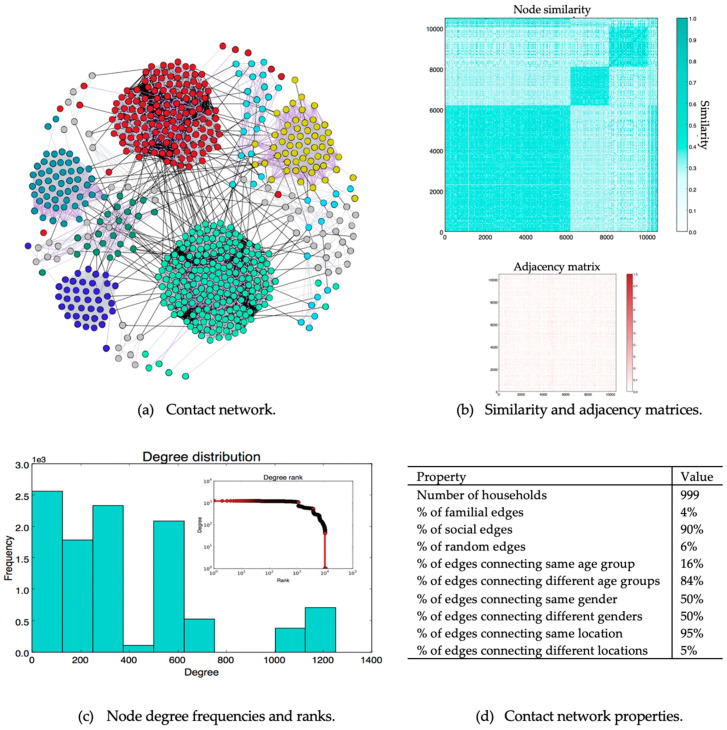
Generated contact network information. (**a**) Part of the generated contact network showing contact relationships between 50 randomly selected households. The gray edges are familial, the purple edges are social, and the black edges are random. (**b**) Similarity and adjacency matrices. Each cell in the adjacency matrix indicates the existence (dark cell) or the absence (white cell) of an edge between each node pair. (**c**) Degree distribution. (**d**) General network properties.

To make the model more realistic, a set of random edges is removed from the network (for example, not all familial relationships resemble infection-leading forms of contact) based on the edge type. A familial edge is removed with a probability of *p*^−^*_familial_*, a social edge is removed with a probability of *p*^−^*_social_*, and a random edge is removed with a probability of *p*^−^*_random_* such that *p*^−^*_familial_*<< *p*^−^*_social_* << *p*^−^*_random_*. Algorithm 1 shows the contact network generation algorithm. Figure 2 shows the main properties of the contact network used in the simulation.
**Algorithm 1** Contact network generation1:Create household clusters (complete graphs) with given average sizes2:$type\left(e_{uv}\right)\leftarrow familial\;\forall \;e_{uv}\;\in E$3:**for each** pair of non-neighboring nodes *u*, *v*
**do**
4:   **if**
$similarity\left(u,\;v\right)>T$
**then**     {*T* is the node pairs similarity threshold}5:     $E\;\leftarrow E\;\cup \;e_{uv}$ with probability $p_{social}^{+}$
6:          $type\left(e_{uv}\right)\leftarrow social$7:   **else**8:      **if**
$location\left(u\right)=location\left(v\right)$ then9:         $E\;\leftarrow E\;\cup \;e_{uv}$ with probability $p_{loc}^{+}$
10:         $type\left(e_{uv}\right)\leftarrow random$11:      **else**
12:         $E\;\leftarrow E\;\cup \;e_{uv}$ with probability $p_{random}^{+}$
13:         $type\left(e_{uv}\right)\leftarrow random$14:**for each**$e_{uv}$ do15:   **if**
$type\left(e_{uv}\right)=familial$ then 16:     $E\;\leftarrow E-\;e_{uv}$ with probability $p_{familial}^{-}$
17:   else 18:     **if**
$type\left(e_{uv}\right)=social$ then 19:         $E\;\leftarrow E-\;e_{uv}$ with probability $p_{social}^{-}$
20:     **else**21:         $E\;\leftarrow E-\;e_{uv}$ with probability $p_{random}^{-}$


Each of the square-shaped regions in the similarity matrix in Figure 2b is formed because of the citizenship attribute. The bottom-left region corresponds to Saudi individuals and the other two correspond to non-Saudi individuals. Non-Saudi individuals are partitioned into two groups because two patterns of contact have been identified between non-Saudi individuals. Due to the model’s stochasticity, similarity alone does not control edge formation (see the adjacency matrix in Figure 2b.

Table 1 lists the structural properties of the underlying contact graph, which may have a significant impact on the dynamics of the disease [66]. The network density is zero for a network with no edges and 1 for a network with all possible edges. Our contact network had a density of 0.036, revealing that it is a sparse network with every node connected to every other node (number of connected components is one).

The node degree is the number of contacts an individual node has, which provides a quantitative measure of the node’s role in the disease transmission process (Figure 2c). The maximum degree shows the most active node (or nodes) in the network, representing individuals contacting a large number of people, such as sales workers and delivery and taxi drivers in highly populated locations (e.g., Riyadh).

In addition, the network exhibits a small-world property with a high clustering coefficient and a short average path length and diameter. The network also shows a strong community structure. The modularity value [67] ranges between −1 and 1 and is used to measure the quality of communities (higher modularity indicates stronger community structure). Our contact network had 14 communities, each of which corresponded to a location (this is expected from the generation model used to create the network).

Generally, our contact network structure matches the properties of other contact networks [36,68,69]. However, unlike other contact network generation models, we did not assume any network properties in advance [64,70,71,72].

### 3.2. Data

**Figure A2:**
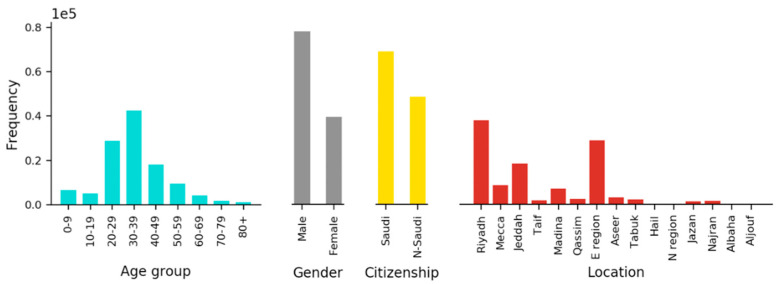
Ratio of individuals per value of all attributes of the COVID-19 patient dataset (patient records from 2 March 2020 to 25 April 2020).

The transmission dynamics of COVID-19 depend on the structure of the underlying contact network and individual susceptibilities. The susceptibility defines how likely an individual is to become infected if he or she comes into contact with an infected individual. Since it is unknown what attributes of an individual determine his or her susceptibility, we used statistical tests on a real COVID-19 patient dataset to identify them. To this end, we requested and received data about the patients in Saudi Arabia from the Saudi Ministry of Health. The data consist of records of all individuals who were tested by taking nasopharyngeal swabs for COVID-19 in Saudi Arabia between 2 March 2020 until 25 April 2020. Several data cleaning steps were applied to the dataset before testing. The characteristics of the final dataset are shown in Figure A2 and Table A2 in the Appendix.

As can be seen in Table A2, the dataset is unbalanced because the majority of the cases are negative. Therefore, we conducted oversampling for the positive class according to the Synthetic Minority Over-sampling Technique (SMOTE) using Python [73]. We then applied the Pearson’s Chi-Square statistical hypothesis test to both the original unbalanced dataset and the balanced dataset. Chi-Square was used to assess whether there was a significant statistical relationship between the attribute (i.e., age, gender, citizenship, and location; the independent variables) and the test result (the dependent variable). This is a well-known feature selection technique in machine learning [74]. Our goal is to determine which attribute contribute to an individual’s susceptibility. The resulting *p*-values for the attributes are presented in Table 2. It can be seen that all *p*-values were < 0.05, which implies a significant relationship. Therefore, all attributes were included to estimate an individual’s susceptibility.

### 3.3. Simulation

Let *G* = (*V,E*) denote the contact network defined in Section 3.1. To simulate the spread of COVID-19 in Saudi Arabia, we ran a standard SIR epidemic model on our contact network. According to this model, each node *u* has a state *state*(*u*) that is either susceptible, infected, or recovered (immune). Transitions are only allowed from susceptible to infected or from infected to recovered. The SIR model is a reasonable representation for COVID-19, which assumes (up to this point) to lead to full immunity after recovery [75].

**Figure A3:**
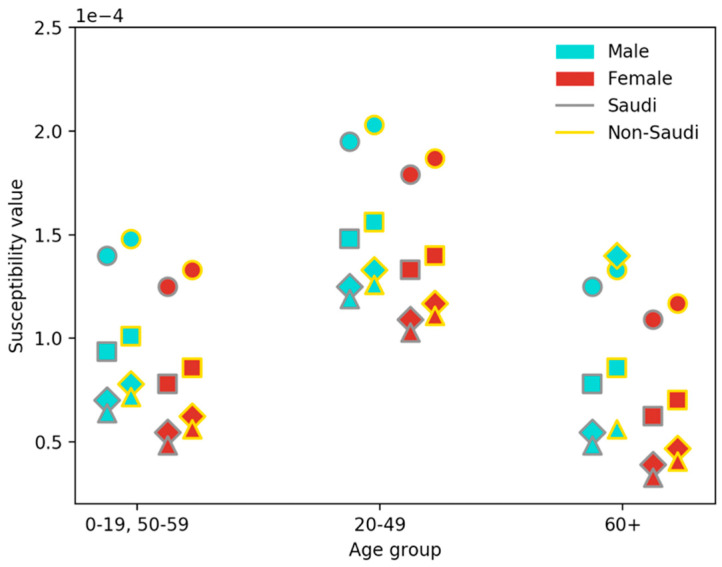
Node susceptibilities based on age group, gender, citizenship, and location. Blue markers represent male nodes and red markers represent female nodes. Marker borders denote citizenship (gray represent Saudi and yellow represent Non-Saudi). Marker shapes denotes the different location as follows. Circles represent Riyadh, Mecca, Jeddah, and the Eastern region. Squares represent Madina. Diamonds represent Taif, Qassim, Aseer, Tabuk, Jazan, and Najran. Triangles represent Hail, Northern region, Albaha, and Aljouf.

The epidemiology of COVID-19 and its clinical characteristics are not fully known. Therefore, we heavily relied on recently available data [54,55] for disease transmission. Based on the analysis in Section 3.2, we identified four main attributes that play a role in the transmission of infection: age, gender, citizenship, and location. Accordingly, each node *u* was assigned a susceptibility value *susceptibility*(*u*) describing its risk of infection. The transmission probability from an infected node *v* to a susceptible node *u* occurs with a probability proportional to the susceptibility of node *u*.; i.e., *p_u,v_* = *susceptibility*(*u*), where *state*(*v*) = *infected*. To find each susceptibility value, we extracted all possible events (attribute value combinations) from the available records and calculated the probability of each compound event. Figure A3 and Table A3 list the node susceptibility values.

Initially (at time 0), the population is fully susceptible with a single infected individual. The infected individual was chosen to have the same attributes (i.e., age group, gender, citizenship, and location) as the first recorded case in Saudi Arabia (a 40-year-old male from the Eastern region). Thereafter, the infection progresses via the contact network for several iterations (each iteration corresponds to one day). The incubation period was set to 14 days, which is the maximum incubation period recorded for COVID-19 [76]. The recovery rate was set to 0.2 (see Table 3).

The major control measures employed by the Saudi government were implemented in the model, which include school closures, mosque closures, domestic flight shutdowns, and in-home curfews. The model also implements social distancing, ground screening, partial business reopening, and business as usual. The major control measures, their dates, and assumed compliance rates used in the model are listed in Table 4. In some cases, control measures are not enough to prevent contact; for example, school friends can meet outside of school, and people can still travel by car to meet. In Table 4, a compliance rate of 35% for ground screening represents the percentage of people who were infected but only detected as a result of the ground screening. Business as usual refers to the full reopening of businesses, where we assume that contact relationships are restored and social distancing is the only measure that affects the susceptibility of individuals. The compliance rates that produced simulation curves closest to the actual curve were selected.

Control measures were introduced by removing edges between relevant nodes and with a specific compliance rate. For example, school closures resulted in removing edges among node pairs who shared the same location and age group. Edges were removed with a specific probability and among a specific percentage of relevant nodes. On the other hand, partial business reopening and business as usual result in adding removed edges between a given set of nodes and with a given probability. Finally, we implemented social distancing as a reduction in the probability of infection (decreasing node susceptibility).

## 4. Results

**Figure 3:**
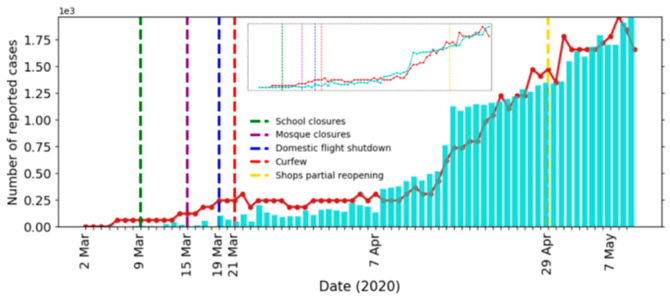
Actual (bar) and simulated (line) number of recorded cases per day. The inset shows the scaled number of new cases.

**Figure 4:**
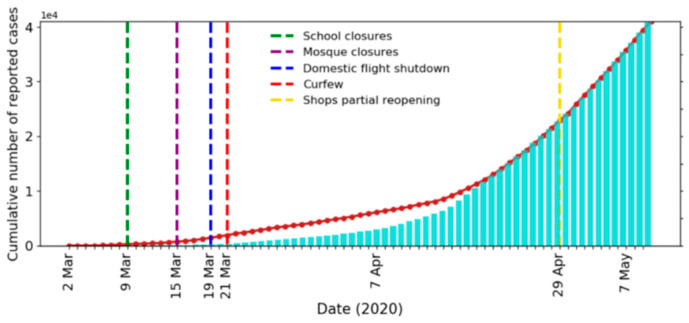
Actual (bar) and simulated (line) cumulative number of recorded cases per day.

To establish the simulation model parameters, we used the empirical data of confirmed cases of COVID-19 in Saudi Arabia for the period from 2 March 2020 (first confirmed case) until 11 May 2020. The model parameters are listed in Table 3. We compare the actual and simulated results of the daily and cumulative new infected cases in Figure 3 and Figure 4, respectively. Note that all simulation results corresponded to averages of 10 simulations and were scaled to the actual number of infected cases.

**Figure 5:**
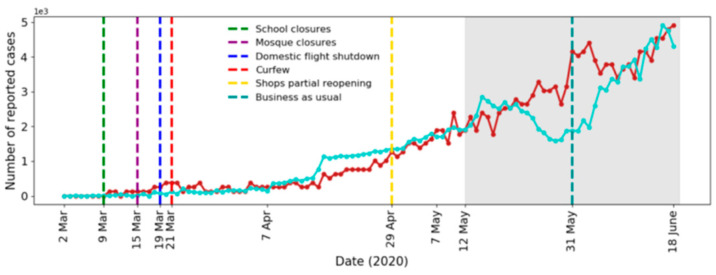
Actual (blue) and simulated (red) number of recorded cases per day. Shaded region: actual (blue) and predicted (red) number of recorded cases for the period 12 May 2020 to 18 June 2020.

It can be seen from the figures that our model fit the reported data well. To further confirm the fit of our model, we predicted the daily cases for the period from 12 May 2020 to 18 June 2020 and compared it with the available actual data (see Figure 5). All regulations imposed after 31 May are not implemented in the model, which may explain the overestimations of the simulated curve around 31 May. The values of the Mean Absolute Percentage Error (MAPE) and the Symmetric Mean Absolute Percentage Error (sMAPE) for the prediction were 17.7% and 14.6%, respectively. Outlier values were removed due to the extreme sensitivity of the above error measures to outliers [77,78]. The values of MAPE and sMAPE before removing the outliers were 32.9% and 19.0%, respectively.

We further simulated and analyzed the effect of the selected Saudi control measures and their timings. Then, we predicted the disease dynamics and measured the effect of vaccination.

### 4.1. Effect of Control Measures

**Figure 6:**
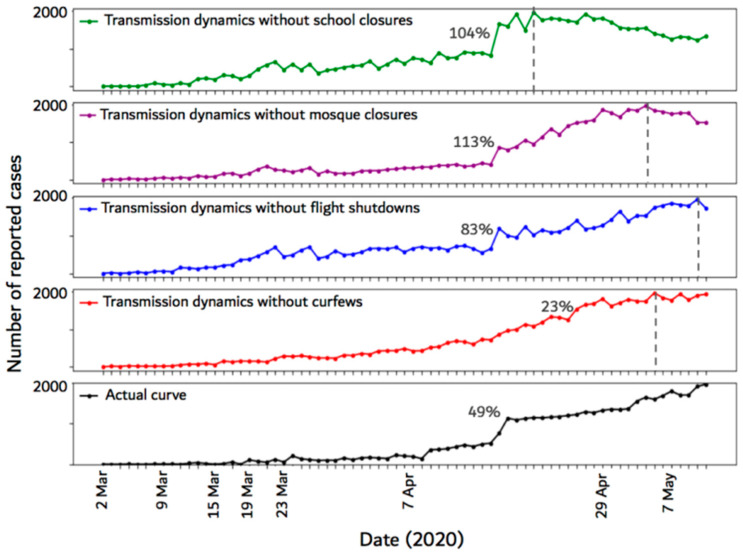
Epidemic curves resulting from not implementing each of the four major control measures employed by the Saudi government. The dashed vertical lines show the time step (day) at which the number of new cases reached the maximum. Each percentage represents the maximum percentage increase in the number of infected cases between any two time-steps on the curve.

To determine the efficacy of the imposed control measures in Saudi Arabia, we simulated the epidemic without each measure individually for the period from 2 March 2020 to 11 May 2020. Then, we compared the resulting simulation curve with the original epidemic curve. The results of this analysis provided an estimate of the number of new cases that were prevented using the control measure. Figure 6 illustrates the epidemic curves produced from not implementing the major control measures (i.e., school closures, mosque closures, domestic flight shutdowns, and curfews) imposed by the Saudi government.

The figure shows that removing any of the control measures caused the epidemic curve to reach the peak earlier compared to the actual curve. When the school closures measure is not implemented, the maximum percentage increase in the number of daily cases was 104% and the curve peak occurred earlier compared to the other curves. Not implementing mosque closures and curfews also caused the curve to peak early compared to the actual curve. Cancelling mosque closures caused 113% maximum percentage increase, while cancelling curfews caused only 23% increase. Cancelling flight shutdowns resulted in 83% maximum percentage increase in the number of daily cases.

### 4.2. Effect of Control Measure Timing

**Figure 7:**
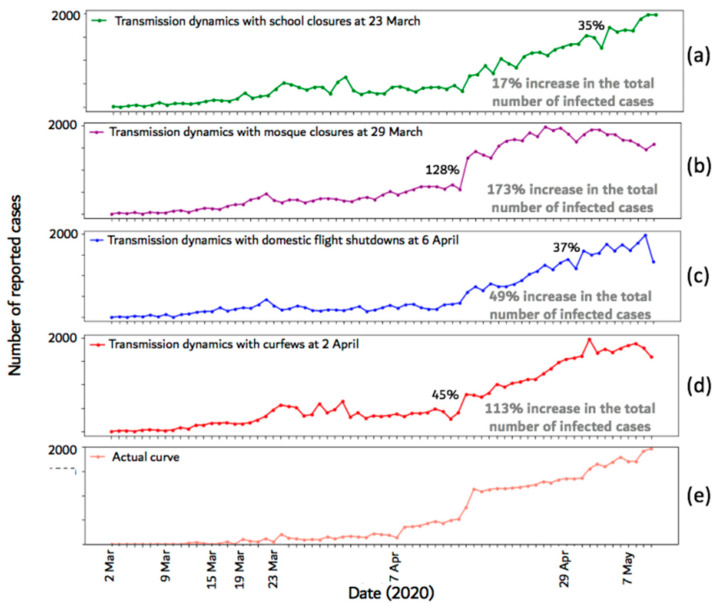
Epidemic curves after changing the effective date of each of the employed control measures. The highest percentage increase in the number of infected cases between any two time-steps and the increase in the total number of infected cases are shown on each plot. (**a**)-(**d**) show the impact of delaying school closures, mosque closures, flight shutdowns, and curfews, respectively. (**e**) shows the actual curve.

To assess the impact of the selected date of each of the control measures, we simulated the epidemic with a late effective date for each measure. The results are shown in Figure 7, where Figure 7a shows the impact of delaying school closures, Figure 7b shows the impact of delaying mosque closures, Figure 7c shows the impact of delaying domestic flight shutdowns, Figure 7d shows the impact of delaying curfews, and Figure 7e shows the actual curve (for ease of comparison). Each figure also shows the percentage increase in the infection rate and the total number of infected cases when the corresponding control measure was delayed.

The figure shows that delaying any of the control measures caused an increase in the infection rate and in the total number of infected cases. When the mosque closures measure was delayed, the total number of infected cases increased by 173% and the infection rate increased by 128%. Delaying curfews caused 113% increase in the total number of infected cases and 45% increase in the infection rate. When school closures or flight shutdowns were delayed, the infection rate increased by 35% and 37%, respectively. Delaying school closures and flight shutdowns increased the total number of infected cases by 17% and 49%, respectively.

### 4.3. Outbreak Prediction

**Figure 8:**
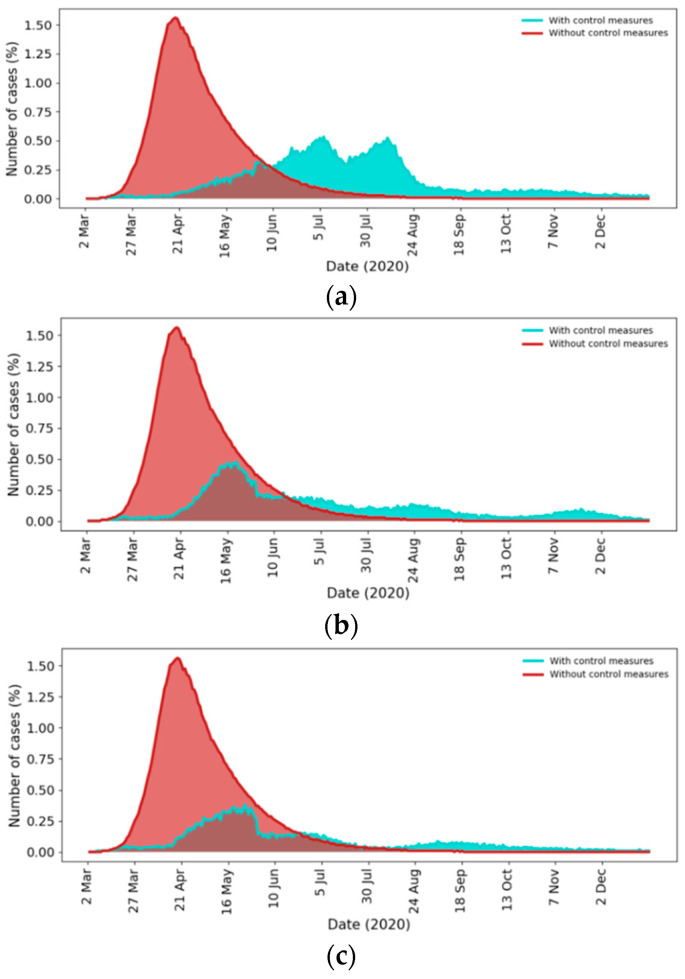
Prediction of future outbreak dynamics with (blue) and without (red) control measures. (**a**) Poor compliance to social distancing; (**b**) Moderate compliance to social distancing; (**c**) Strong compliance to social distancing.

We used the proposed model to predict the future dynamics of the outbreak in Saudi Arabia for the upcoming period of six months (From 12 May to 31 December) with respect to three scenarios, representing multiple levels of adherence to the social distancing recommendations after 31 May 2020 (the announced business as usual date). Figure 8 shows the number of infected individuals per day for all scenarios. In particular, Figure 8a–c show the epidemic dynamics with poor (0% of population), moderate (50% of population), and strong (75% of population) compliance to social distancing, respectively. The level of adherence is defined by the percentage of people that practice social distancing. For comparison purposes, we also predicted the dynamics of the outbreak when no control measures or social distancing were imposed during the whole pandemic period starting from 2 March.

The red curve in Figure 8 shows that if no control measures were imposed, the peak of the infection was predicted to be about 1.6% on 21 April 2020 with a total outbreak size of 80% of the population (the peak refers to the highest number of daily infections) and the epidemic ended at the end of August 2020.

Figure 8a shows the disease dynamics when social distancing adherence is poor (0% of the population is practicing social distancing) after business return on 31 May 2020. The simulation results suggest that there would be two peaks at roughly the beginning of July and the middle of August. The peak of the infection will be 0.5% with a total outbreak size of about 47% of the population. According to this scenario, our model suggests that the epidemic will end at the beginning of November 2020 with over 13 million infected individuals, which is measured according to the number of active cases (i.e., when the number of active cases is close to zero).

The epidemic curve in Figure 8b shows the disease dynamics when social distancing is practiced moderately (about 50% of the population practicing social distancing). The figure suggests that the first peak will remain below 0.4%, the second peak will be avoided, and the total number of infected individuals will be about 33% of the population. In Figure 8c, the epidemic curve suggests that when most people practice social distancing (75% of the population), the total number of infected individuals will decrease to about 25%.

### 4.4. Effect of Vaccination

**Figure 9:**
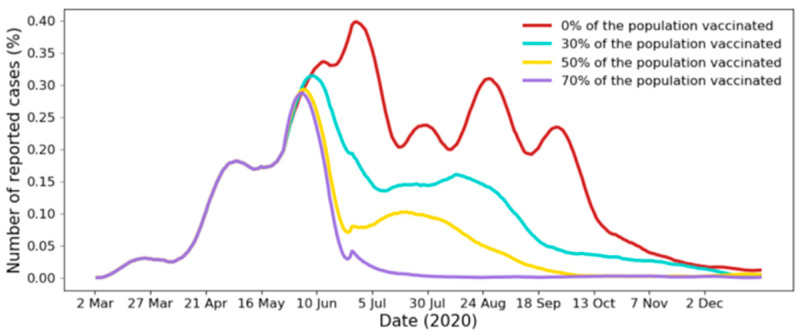
Epidemic curves of multiple vaccination scenarios. Curves are smoothed using a Savitzky-Golay filter [79] (filter with a window length of 31 and a degree 3 polynomial).

We next explored the dynamics of the epidemic if part of the population is vaccinated. This is helpful to understand what percentage of the population must be vaccinated to stop the epidemic. We considered four scenarios where 0%, 30%, 50%, and 70% of the population was vaccinated. In the proposed model, vaccination is represented by removing the edges between a node *u* and part of its neighbor nodes. We show the epidemic curves and the results of multiple vaccination scenarios in Figure 9 and Table 5, respectively. We assumed that a vaccine would become available on 10 June 2020 (the date was chosen to make the differences easily visible on the plot). Before this date, all control measures are imposed with the compliance rates shown in Table 4. However, irrespective of the dates, the insights available in this simulation are useful for whenever a vaccine becomes available.

Figure 9 and Table 5 suggest that the outbreak and peak sizes are inversely proportional to the percentage of vaccinated population. Further, we observe that the higher the percentage of population vaccinated is, the earlier the epidemic peaks and the epidemic ends. For example, when 30% of the population is vaccinated, the peak occurs on 1 July 2020 and ends on 4 November 2020. When 70% of the population is vaccinated, the peak occurs on 30 May 2020 and ends on 25 June 2020.

## 5. Discussion

The proposed network model allows the analysis and evaluation of various control measures that are used to slow or prevent the transmission of COVID-19 in Saudi Arabia and to evaluate the timing of each measure. Moreover, the model can be used to predict the future dynamics of the outbreak in Saudi Arabia with and without the availability of vaccination.

### 5.1. Effect of Control Measures

The results presented in Section 4.1 show the epidemic curves resulting from not implementing each of the four major control measures employed by the Saudi government. The results reveal several important pieces of information. First, they suggest that all of the employed control measures played a significant role in delaying the peak of the epidemic, where the peak represents the highest number of daily infections. This can be seen by comparing the dashed vertical lines on the curves, which show the day at which the number of new cases reached the maximum peak (compared to the actual curve). Secondly, it is apparent from the top curve in Figure 6, that implementing school closures had the maximum impact because canceling school closures caused the curve to reach the peak early compared to the other three curves. Thirdly, the results suggest that the employed measures also played an important role in slowing down the infection rate. For example, the maximum percentage increase in the number of cases in the original curve was 49%, whereas it was 104%, 113%, and 83% without implementing school closures, mosque closures, and flight shutdowns, respectively. Our results are in agreement with previous findings in China [11,24,25] and in the United States [11]. For example, the authors in [11] suggested that community mitigation actions such as isolation of infectious individuals, quarantine of close contacts, and travel restrictions impact the COVID-19 disease infection rates.

Canceling curfews appeared to have a minimum impact on slowing down the infection rate compared to other measures as it only caused a 23% increase in the number of cases (red curve in Figure 6). This is likely because the assumed compliance rate for this measure was only 50% (see Table 4), which is the lowest compared to those of other measures.

### 5.2. Effect of Control Measure Timing

The time at which the control measures are implemented against the epidemic is critical. In Section 4.2, we presented the epidemic curves after changing the effective date of each of the employed control measures. From the results, it can be observed that delaying one of the measures increased the total number of infected cases compared to when all measures were implemented (the actual curve). For instance, when the effective date of the school closure control measure was delayed by 14 days, the total number of infected cases increased by 17%. However, the highest increase in the number of infected cases was seen when mosque closures and curfews were delayed (173% and 113%, respectively).

Similar trends can be observed with respect to the infection rate as delaying mosque closures and curfew implementation caused maximum percentage increases (128% and 45%, respectively). This can be explained by the susceptibility of the nodes affected by each control measure. For example, mosque closures mainly affect contact relationships (edges) among adult males. Generally, the node susceptibilities of adult male individuals are higher compared to those of other nodes (Table A3 and Section 3.2). Delaying flight shutdowns had the least impact on the total number of infected cases (4% increase compared to the actual). This can be explained by the low number of edges that connect individuals from different locations (Figure 2(d)).

### 5.3. Outbreak Prediction

The model was used to predict the future dynamics of COVID-19 in Saudi Arabia for the upcoming period of six months with and without control measures. The results are shown in Section 4.3. A number of observations can be made from the results. First, the size of the peak when no control measures were imposed would be disastrous as it would result in a total of over 26 million infected individuals, which would overwhelm the healthcare system.

We next compare the three scenarios when control measures are imposed. First, if social distancing adherence is poor, the two peaks, occurring at roughly the beginning of July and the middle of August, would result in over 13 million infected individuals by the end of the epidemic (at the beginning of November 2020). This number is half of the number of infected individuals when no control measures were imposed. Second, social distancing significantly decreased the infection peak and the total number of infections. Our results contradict earlier findings [20,21,80,81]. For example, [80] predicted that the 99% pandemic end in Saudi Arabia should have been on 30 May 2020. In [20], the authors predicted that the final phase of the outbreak would occur by the end of June 2020 with a total of 79,000 infected individuals. Further, the work in [21] predicted that pandemic would end by early September 2020 with a total of 359,794 infected individuals.

In comparing the two scenarios (without control measures and with control measures), it is clear that employing different control measures is crucial for flattening the epidemic curve and reducing the final size of the epidemic. These measures also prolong the peak period and minimize the peak, which is crucial to avoid overwhelming the healthcare system.

### 5.4. Effect of Vaccination

The model was used to predict the disease dynamics under multiple vaccination scenarios. The results (Section 4.4) suggest that the epidemic will end in Saudi Arabia on 4 November 2020 if no one in the population is vaccinated (i.e., if no vaccination is available). At this point, a sufficient amount of the population developed immunity to the disease because they previously had the virus and had recovered.

However, the results showed that, in this scenario, around 41% of the population may become infected, which is equivalent to over 13 million individuals, and the epidemic may reach its peak on 1 July 2020 with over a hundred thousand individuals infected. In the best-case scenario, when 70% of the population is vaccinated on 10 June the results suggested that it may take only 15 days to end the epidemic with an outbreak size of 13%. This period increases by almost two months when only 50% of the population is vaccinated. When 30% of the population is vaccinated, the results show that the epidemic may end in late September. Note that specific recommendations for vaccination may consider multiple factors analyzed in this section, such as the outbreak size, peak size, and pandemic end date, but may also consider other factors, such as the vaccination cost and the number of critical cases.

### 5.5. Limitations

The proposed network generation and simulation models are part of an effort to create an accurate simulation of the spread of COVID-19 in Saudi Arabia. However, the findings in this work are subject to several limitations. As with all models, the quality of our model depends on the quality of the underlying data. This includes the contact patterns, data of infected cases, and pathogen data. Inadequate and missing data were replaced with assumptions and simplifications. For example, in the proposed contact network generation model, the contact patterns and edge formation among individuals were simplified to three contact types with corresponding assumed probabilities. Therefore, a greater focus on realistic contact patterns in Saudi Arabia using a social contact survey could produce interesting findings that could enhance the accuracy of our model.

Moreover, the contact network used to simulate the disease was static. A dynamic network, in which nodes and edges are added and removed over time due to birth, death, and quarantine, would be more realistic to represent contact relationships among individuals; this is left for future work. Our predictions also include inherent uncertainty as the model parameters were derived from limited clinical data. For example, the node susceptibilities were based on limited data (records from 2 March to 25 April). In addition, the population’s actual compliance to the recommended control measures is unknown. Therefore, the compliance rates used in the model were assumed. More information on population compliance rates would help improve the accuracy of the model.

## 6. Conclusions

The goal of this work was to model and analyze the spread of COVID-19 in Saudi Arabia using a network-based epidemic model. First, we generated a realistic contact network of individuals in Saudi Arabia. Then, we used the SIR model to simulate the spread of COVID-19. The proposed model accounted for the dynamic nature of individual contact behaviors and the variations in susceptibility between individuals. The proposed simulation model was used to evaluate the effectiveness of the employed Saudi control measures and their timings on the dynamics of the epidemic and to predict the future dynamics of the outbreak in Saudi Arabia. The model was also used to calculate the percentage of people that need to be vaccinated to stop the epidemic.

## Figures and Tables

**Table 1:** Contact network properties.

Property	Definition	Value
Number of nodes	Number of individuals in the contact network	10,500
Number of edges	Number of connections between individuals in the contact network	1,994,082
Network density	Ratio of the number of edges to the number of possible edges.	0.036
Number of connected components	Parts of the network in which all nodes are connected	1
Node degree	Number of edges connected to a node	
Average degree	Average number of edges per node	380
Maximum degree	Degree of nodes with the greatest number of edges	1252
Minimum degree	Degree of nodes with the smallest number of edges	1
Network diameter	Length of longest shortest path over all node pairs	5
Network average path length	Over all shortest paths connecting node pairs	2.34
Network clustering coefficient	Extent to which neighbors of a node to form connections.	0.25
Network Community structure	Degree to which nodes can be grouped into internally dense sets	
Modularity		0.62
Number of communities		14

**Table 2:** Chi-square *p*-values for different individual attributes.

Type	Attribute	*p*-Value
Unbalanced data	Age	2.21775012 × 10^−020^
	Gender	4.73960493 × 10^−087^
	Citizenship	0.00000000 × 10^+000^
	Location	0.00000000 × 10^+000^
Balanced data	Age	4.57937250 × 10^−274^
	Gender	3.62576524 × 10^−121^
	Citizenship	0.00000000 × 10^+000^
	Location	0.00000000 × 10^+000^

**Table 3:** Simulation model parameters.

Symbol	Value	Description	Type
*T*	0.7	Lower limit of the similarity between two nodes	Threshold
*p* ^+^_social_	0.25	Probability of connecting a pair of similar nodes	Node pair connection ratio
*p* ^+^_loc_	0.15	Probability of connecting a pair of non-similar nodes in the same location	Node pair connection ratio
*p* ^+^_random_	0.01	Probability of connecting a pair of non-similar nodes in different locations	Node pair connection ratio
*p* ^−^_familial_	0.0001	Probability of deleting a familial edge connecting a pair of nodes	Edge deletion ratio
*p* ^−^_social_	0.005	Probability of deleting a social edge connecting a pair of nodes	Edge deletion ratio
*p* ^−^_random_	0.001	Probability of deleting a random edge connecting a pair of nodes	Edge deletion ratio
*τ*	14 days	Incubation period	Threshold
*γ*	0.2	Rate of transition from the infected state to the recovered state	Recovery rate

**Table 4:** Major control measures employed by the Saudi government during the COVID-19 pandemic. The effective dates and assumed compliance rates are listed as assumed in our model.

Measure	Effective Date	Compliance Rate
School closures	9 March 2020	95%
Mosque closures	15 March 2020	65%
Domestic flight shutdowns	21 March 2020	85%
Curfews	23 March 2020	50%
Ground screening	10 April 2020	35%
Partial business reopening	29 April 2020	50%
Business as usual	31 May 2020	-

**Table 5:** Epidemic curves of multiple vaccination scenarios.

Percentage of Population Vaccinated	Peak Size (% of Population)	Peak Date	Outbreak Size	End (95%)	End (99%)
0%	0.39%	1 July	41%	4 November	-
30%	0.32%	9 June	31%	24 September	9 December
50%	0.28%	1 June	19%	30 August	27 September
70%	0.27%	30 May	13%	25 June	17 July

## Appendix

**Table A1:** Distribution of nodes by age group, gender, citizenship, and location. The age distribution of individuals was based on citizenship and gender but is approximated here. Similarly, the gender distribution was also based on citizenship but is approximated here.

Attribute	Values	Percent
Age group	0–9	15%
	10–19	15%
	20–29	20%
	30–39	20%
	40–49	15%
	50–59	8%
	60–69	4%
	70–79	2%
	80+	1%
Gender	Male	50%
	Female	50%
Citizenship	Saudi	62%
	Non Saudi	38%
Location	Riyadh	25%
	Mecca	8%
	Jeddah	14%
	Taif	3%
	Madina	7%
	Qassim	4%
	Eastern region	15%
	Aseer	7%
	Tabuk	3%
	Hail	2%
	Northern region	1%
	Jazan	5%
	Najran	2%
	Albaha	2%
	Aljouf	2%

**Table A2:** Attributes of the COVID-19 patient dataset (patient records from 2 March 2020 to 25 April 2020).

Attribute	Value	Number of Individuals
Data size		117,840
Age	0–9	6601
	10–19	5123
	20–29	28,785
	30–39	42,506
	40–49	18,226
	50–59	9382
	60–69	4232
	70–79	1813
	80+	1172
Gender	Male	78,197
	Female	39,643
Citizenship	Saudi	69,126
	Non-Saudi	48,715
Location	Riyadh	38,142
	Mecca	8813
	Jeddah	18,486
	Taif	2028
	Madina	7270
	Qassim	2675
	Eastern region	28,971
	Aseer	3249
	Tabuk	2420
	Hail	117
	Northern region	500
	Jazan	1502
	Najran	1711
	Albaha	454
	Aljouf	502
Test result	Positive	9855
	Negative	107,985

**Table A3:** Simulation model parameters: node susceptibilities based on attributes.

Gender	Citizenship	Age Group	Location	Node Susceptibility Value
Male	Saudi	0–19, 50–59	Riyadh, Mecca, Jeddah, Eastern Reg.	1.40 × 10^−04^
			Madina	9.36 × 10^−05^
			Taif, Qassim, Aseer, Tabuk, Jazan, Najran	7.02 × 10^−05^
			Hail, Northern Reg., Albaha, Aljouf	6.40 × 10^−05^
		20–49	Riyadh, Mecca, Jeddah, Eastern Reg.	1.95 × 10^−04^
			Madina	1.48 × 10^−04^
			Taif, Qassim, Aseer, Tabuk, Jazan, Najran	1.25 × 10^−04^
			Hail, Northern Reg., Albaha, Aljouf	1.19 × 10^−04^
		≥60	Riyadh, Mecca, Jeddah, Eastern Reg.	1.25 × 10^−04^
			Madina	7.80 × 10^−05^
			Taif, Qassim, Aseer, Tabuk, Jazan, Najran	5.46 × 10^−05^
			Hail, Northern Reg., Albaha, Aljouf	4.84 × 10^−05^
	Non-Saudi	0–19, 50–59	Riyadh, Mecca, Jeddah, Eastern Reg.	1.48 × 10^−04^
			Madina	1.01 × 10^−04^
			Taif, Qassim, Aseer, Tabuk, Jazan, Najran	7.80 × 10^−05^
			Hail, Northern Reg., Albaha, Aljouf	7.18 × 10^−05^
		20–49	Riyadh, Mecca, Jeddah, Eastern Reg.	2.03 × 10^−04^
			Madina	1.56 × 10^−04^
			Taif, Qassim, Aseer, Tabuk, Jazan, Najran	1.33 × 10^−04^
			Hail, Northern Reg., Albaha, Aljouf	1.26 × 10^−04^
		≥60	Riyadh, Mecca, Jeddah, Eastern Reg.	1.33 × 10^−04^
			Madina	8.58 × 10^−05^
			Taif, Qassim, Aseer, Tabuk, Jazan, Najran	24 × 10^−05^
			Hail, Northern Reg., Albaha, Aljouf	5.62 × 10^−05^
Female	Saudi	0–19, 50–59	Riyadh, Mecca, Jeddah, Eastern Reg.	1.25 × 10^−04^
			Madina	7.80 × 10^−05^
			Taif, Qassim, Aseer, Tabuk, Jazan, Najran	5.46 × 10^−05^
			Hail, Northern Reg., Albaha, Aljouf	4.84 × 10^−05^
		20–49	Riyadh, Mecca, Jeddah, Eastern Reg.	1.79 × 10^−04^
			Madina	1.33 × 10^−04^
			Taif, Qassim, Aseer, Tabuk, Jazan, Najran	1.09 × 10^−04^
			Hail, Northern Reg., Albaha, Aljouf	1.03 × 10^−04^
		≥60	Riyadh, Mecca, Jeddah, Eastern Reg.	1.09 × 10^−04^
			Madina	6.24 × 10^−05^
			Taif, Qassim, Aseer, Tabuk, Jazan, Najran	3.90 × 10^−05^
			Hail, Northern Reg., Albaha, Aljouf	3.28 × 10^−05^
	Non-Saudi	0–19, 50–59	Riyadh, Mecca, Jeddah, Eastern Reg.	1.33 × 10^−04^
			Madina	8.58 × 10^−05^
			Taif, Qassim, Aseer, Tabuk, Jazan, Najran	6.24 × 10^−05^
			Hail, Northern Reg., Albaha, Aljouf	5.62 × 10^−05^
		20–49	Riyadh, Mecca, Jeddah, Eastern Reg.	1.87 × 10^−04^
			Madina	1.40 × 10^−04^
			Taif, Qassim, Aseer, Tabuk, Jazan, Najran	1.17 × 10^−04^
			Hail, Northern Reg., Albaha, Aljouf	1.11 × 10^−04^
		≥60	Riyadh, Mecca, Jeddah, Eastern Reg.	1.17 × 10^−04^
			Madina	7.02 × 10^−05^
			Taif, Qassim, Aseer, Tabuk, Jazan, Najran	4.68 × 10^−05^
			Hail, Northern Reg., Albaha, Aljouf	4.06 × 10^−05^
